# Incidence, associated outcomes, and predictors of upper gastrointestinal bleeding following acute myocardial infarction: a SWEDEHEART-based nationwide cohort study

**DOI:** 10.1093/ehjcvp/pvab059

**Published:** 2021-08-23

**Authors:** Philip Sarajlic, Moa Simonsson, Tomas Jernberg, Magnus Bäck, Robin Hofmann

**Affiliations:** Department of Medicine, Karolinska Institute, Stockholm, Sweden; Department of Clinical Sciences, Danderyd University Hospital, Karolinska Institute, Stockholm, Sweden; Theme Heart and Vessels, Division of Valvular and Coronary Disease, Karolinska University Hospital, Stockholm, Sweden; Department of Clinical Sciences, Danderyd University Hospital, Karolinska Institute, Stockholm, Sweden; Department of Medicine, Karolinska Institute, Stockholm, Sweden; Theme Heart and Vessels, Division of Valvular and Coronary Disease, Karolinska University Hospital, Stockholm, Sweden; Department of Clinical Science and Education, Division of Cardiology, Karolinska Institute, Sodersjukhuset, Stockholm, Sweden

**Keywords:** Upper gastrointestinal bleeding, Predictors, Acute myocardial infarction, Registry

## Abstract

**Aims:**

Of all spontaneous bleeding complications in patients with acute myocardial infarction (MI), upper gastrointestinal bleeding (UGIB) is common and of specific interest since it could be prevented by several prophylactic measures. We aimed to determine the incidence, associated outcomes, and predictors of UGIB following acute MI.

**Methods and results:**

All patients with acute MI enrolled in the SWEDEHEART (Swedish Web-system for Enhancement and Development of Evidence-based care in Heart disease Evaluated According to Recommended Therapies) registry from January 2007 to June 2016 and discharged alive on any antithrombotic therapy (*n* = 149 477) were followed regarding UGIB for 1 year. Associated outcomes were determined by Cox proportional hazards regression with UGIB as a time-dependent covariate, adjusting for baseline characteristics, invasive treatment, and medical treatment at discharge. Predictors of UGIB were determined by logistic regression and machine learning models.

At 1 year, UGIB had occurred in 2230 patients (cumulative incidence 1.5%) and was significantly associated with an increased risk of all-cause death [hazard ratio (HR) 2.86, 95% confidence interval (CI) 2.58–3.16] and stroke (HR 1.80, 95% CI 1.32–2.45) but not with recurrent MI (HR 1.17, 95% CI 0.97–1.42). The most important predictors of UGIB were haemoglobin, age, systolic blood pressure, blood glucose, smoking status, previous upper gastrointestinal bleeding, and antithrombotic and gastroprotective treatment.

**Conclusion:**

After acute MI, readmission because of UGIB is common and significantly associated with poor prognosis. By using machine learning in addition to traditional logistic regression, new predictors of UGIB, such as blood glucose and smoking status, were identified.

## Graphical Abstract

**Graphical Abstract:**
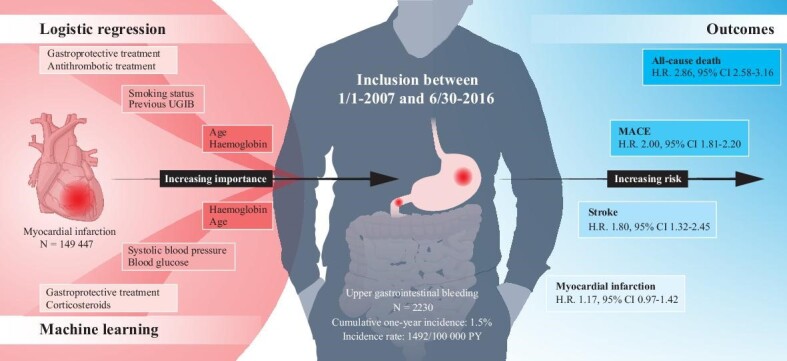
Incidence, predictors, and associated outcomes following acute myocardial infarction.

## Introduction

Dual antiplatelet therapy (DAPT) with aspirin and one P2Y12 inhibitor is the default antithrombotic strategy after acute myocardial infarction (MI) irrespective of invasive or conservative treatment.^1,2^ This strategy improves ischaemic outcomes but is counterbalanced by an increased risk of bleeding. In the last few decades, the prognostic importance of bleeding events has been well established as several studies have shown a strong association between bleeding and mortality.^3^ The goal of future antithrombotic strategies is now beyond only ischaemic protection but also focused on bleeding reduction.^4^

The most common location of spontaneous, non-access site bleeding is the gastrointestinal tract.^5^ Of these, upper gastrointestinal bleeding (UGIB) is common and of special interest, since it may to some extent be prevented by, for example, prophylactic use of proton pump inhibitors (PPIs),^6,7^ aspirin-free strategies,^8,9^ or *Helicobacter pylori* eradication.^10^ The European Society of Cardiology (ESC) recommends PPIs in patients with higher-than-average risk of gastrointestinal (GI) bleeding defined as a history of gastric ulcer/bleeding, anticoagulant therapy, chronic non-steroidal anti-inflammatory drug (NSAID)/corticoid steroid use, or two or more of age ≥65 years, dyspepsia, gastro-oesophageal reflux disease, *H. pylori* infection, or chronic alcohol use.^2^

Currently, predictors and associated cardiovascular outcomes of UGIB after acute MI are not sufficiently understood. First, available data are derived from smaller studies with selected patient populations commonly including all types of GI bleeding^11,12^ and data from larger unselected MI populations are scarce. Second, when exploring predictors, traditional risk prediction with logistic regression may miss important aspects due to inferior performance with regards to complex and/or non-linear relationships.

Thus, by using comprehensive data from multiple compulsory national registries, our aims were (i) to determine the 1-year incidence of UGIB, (ii) to establish ischaemic outcomes associated with UGIB, and (iii) to identify the strongest predictors of UGIB in patients with acute MI. For the last aim, we used two different approaches: traditional logistic regression including variables based on previous knowledge and machine learning (ML) including all available data of potential interest.

## Methods

### Data sources

We analysed data from compulsory Swedish national registries linked to the SWEDEHEART (Swedish Web-system for Enhancement and Development of Evidence-based care in Heart disease Evaluated According to Recommended Therapies) registry^13^ that collects information on baseline characteristics, in-hospital course and treatment, and medication on arrival, in hospital, and at discharge from all Swedish coronary care units (*n* = 72). The diagnosis of MI is determined by the responsible physician according to current guidelines. The registry is monitored regularly, showing a 95–96% agreement between key variables and electronic health records. According to Swedish law, no written informed consent is required, but all patients are informed of their participation in the registry and that they have the right to opt out.

The National Patient Registry (NPR) includes all International Classification of Diseases (ICD) codes for all hospital admissions since 1987 and outpatient specialist visits since 2001 but does not cover general practitioners.^14^ The national Prescribed Drug Registry (PDR) registers all dispensed drugs from pharmacies in the country, including variables such as type of medication, dose, prescription date, and dispensation date. The Swedish population registry holds information on life events with complete coverage on death events.

The National Board of Health and Welfare approved the merging of the registries, and the study has been granted approval by the ethics committee in Stockholm (2014/1484-32, 2015/332-32). The data underlying this article cannot be shared publicly due to legal reasons.

### Study population

All patients admitted with acute MI (defined as ICD-10, I21, or I22) enrolled in the SWEDEHEART registry and discharged alive from January 2007 until June 2016 were included. Patients younger than 18 years or discharged without any antithrombotic therapy were excluded. For patients admitted more than once during the study period, only the first admission was included.

### Outcome and variable definitions

UGIB at 1 year was defined as any rehospitalization with a UGIB ICD-10 code (Supplementary material online, *Table S1*) as a primary or secondary diagnosis in the NPR. MI at 1 year was defined as rehospitalization with acute MI in the SWEDEHEART registry (days 2–30) or in the NPR (after day 30) with ICD code I21 as a primary or secondary diagnosis. Ischaemic stroke at 1 year was defined as rehospitalization with ICD-10 code I63 as a primary or secondary diagnosis in the NPR. All-cause death was captured from the Swedish population registry. Major adverse cardiovascular event (MACE) at 1 year was defined as a composite of MI, stroke, and all-cause death. Antithrombotic treatment at discharge was captured from the SWEDEHEART registry. Other medications at discharge—gastroprotective treatment [Anatomical Therapeutic Chemical (ATC) code A02B], corticosteroids (ATC codes H02A and H02B), and NSAIDs (ATC code M01A)—were defined as any outtake 6 months before and/or up to 2 weeks after discharge in the PDR. Gastroprotective drugs included all drugs with indication for gastric ulcer or gastro-oesophageal reflux disease comprising both PPIs and histamine-2 receptor antagonists.

Previous bleeding was defined as any hospitalization with an ICD code of bleeding (Supplementary material online, *Table S2A*) in the NPR before the index MI. Previous UGIB was defined as any hospitalization with an ICD code of UGIB (Supplementary material online, *Table S2B*) in the NPR before the index MI.

### Statistical analysis

Continuous variables are presented as medians (interquartile range). Categorical variables are presented as counts and percentages. Unadjusted incidence of MACE is illustrated graphically using the Simon–Makuch method in which UGIB is considered as a time-dependent event. Logistic regression with UGIB as the outcome and 25 predictor variables {haemoglobin, sex, age, weight, ST-segment elevation myocardial infarction (STEMI), in-hospital coronary angiography, in-hospital percutaneous coronary intervention (PCI), in-hospital coronary artery bypass grafting (CABG), smoking status, hypertension, diabetes, previous MI, previous PCI, previous CABG, previous heart failure, previous stroke, previous lower extremity artery disease, previous UGIB, previous cancer, previous chronic obstructive pulmonary disease, creatinine, C-reactive protein, gastroprotective treatment, corticosteroid treatment, NSAID treatment, and antithrombotic treatment including the five categories: single antiplatelet therapy (SAPT), oral anticoagulant (OAC) alone, DAPT clopidogrel, DAPT ticagrelor/prasugrel, and combination therapy [antiplatelet therapy (APT) + OAC]} selected based on previous knowledge and clinical relevance was performed. Predictor importance was assessed by ranking of the Wald $\chi $^2^ value. Cox proportional hazards regression with MACE and with the individual components (MI, stroke, and all-cause death) as the outcome was performed. All Cox models were adjusted for baseline characteristics, in-hospital treatment, and medication at discharge (Supplementary material online, *Table S3*). Restricted cubic splines with three knots at the 10th, 50th, and 90th percentiles were used to model continuous variables in the regression models. Hazard ratios (HRs) for the categorical predictors are presented with 95% confidence intervals (CIs). The continuous predictors are presented graphically as the estimated spline transformation vs. log odds of UGIB. All significance analyses were two-tailed, and the alpha level was set at 0.05.

Assuming a missing-at-random mechanism, missing data were handled by *k*-nearest neighbour (*k*-NN) imputation where the weighted mean was used to substitute values that were missing. The *k*-NN imputation algorithm was chosen because it can impute both numerical and categorical variables and preserves the data structure and variable distributions of the original dataset. Statistical analyses were conducted in R version 4.0.3.

To further explore the association between clinical variables collected in the SWEDEHEART patient cohort and UGIB, we trained and validated four ML models predicting bleeding events from 105 candidate variables (Supplementary material online, *Table S4*). Variable importance (shown as weights) for included predictors was calculated for the model with the highest performance measured after 10-fold cross-validation. Model performance was assessed through comparison of receiver operating characteristic curves. All ML models were created and assessed in RapidMiner Studio 9.8 (RapidMiner, Inc., 2020) as previously described.^15^ Further details regarding the training and validation of the ML models can be found in the Supplementary material online, *Appendix*.

## Results

### Baseline characteristics and incidence of upper gastrointestinal bleeding

Between 1 January 2007 and 30 June 2016, 149 447 patients were admitted with acute MI and discharged alive on any antithrombotic therapy (Supplementary material online, *Figure S1*). UGIB at 1 year occurred in 2230 patients with a cumulative incidence of 1.5% and an incidence rate of 1492 cases per 100 000 person-years.

Baseline characteristics for patients with or without UGIB at 1 year are summarized in *Table 1*. Patients with a UGIB event were older, more often female, more often previous or current smokers, and had an overall higher burden of comorbidities except for previous revascularization that was equally distributed between the two groups. Previous UGIB was more than three times as common (7.6% vs. 2.0%) in patients with a UGIB event. Treatment with anticoagulant, either as single therapy or in combination with APT, as well as treatment with steroids or NSAIDs, was more common in the UGIB event group, while DAPT was less often prescribed in this group. Any gastroprotective treatment was more common in patients with UGIB (41.0% vs. 28.3%).

**Table 1:** Baseline characteristics

	No UGIB	UGIB
	(*n* = 147 217)	(*n* = 2230)
*Demographics*		
Age, years, median (IQR)	71 (62–80)	77 (68–83)
Female sex, *n* (%)	51 292 (34.8)	861 (38.6)
Weight, kg, median (IQR)	78 (69–89)	76 (65–86)
STEMI, *n* (%)	48 973 (33.3)	662 (29.7)
Smoking status		
Never, *n* (%)	66 998 (45.5)	904 (40.5)
Former, *n* (%)	49 590 (33.7)	825 (37.0)
Active, *n* (%)	30 629 (20.8)	501 (22.5)
*Medical history*		
Hypertension, *n* (%)	82 397 (56.0)	1495 (67.0)
Diabetes, *n* (%)	35 769 (24.3)	617 (27.7)
Previous MI, *n* (%)	32 783 (22.3)	536 (24.0)
Previous PCI, *n* (%)	17 707 (12.0)	265 (11.9)
Previous CABG, *n* (%)	11 824 (8.0)	185 (8.3)
Previous HF, *n* (%)	14 802 (10.1)	354 (15.9)
Previous stroke, *n* (%)	17 070 (11.6)	340 (15.2)
Previous LEAD, *n* (%)	8117 (5.5)	211 (9.5)
Previous UGIB, *n* (%)	2951 (2.0)	170 (7.6)
Previous cancer, *n* (%)	4381 (3.0)	126 (5.7)
Previous COPD, *n* (%)	10 688 (7.3)	276 (12.4)
*Laboratory parameters*		
Haemoglobin, g/L, median (IQR)	138 (126–149)	129.0 (117–142)
Creatinine, mmol/L, median (IQR)	84.0 (70.0–102.0)	90.0 (73.0–117.6)
CRP, mg/L, median (IQR)	6.0 (3.0–17.9)	9.0 (4.0–29.0)
*Invasive treatment in hospital*		
PCI, *n* (%)	90 630 (61.6)	1252 (56.1)
CABG, *n* (%)	6093 (4.1)	73 (3.3)
*Medication at discharge*		
Gastroprotective treatment, *n* (%)	41 617 (28.3)	914 (41.0)
Corticosteroid, *n* (%)	5182 (3.5)	137 (6.1)
NSAID, *n* (%)	2499 (1.7)	49 (2.2)
*Antithrombotic treatment*		
SAPT, *n* (%)	24 180 (16.6)	421 (19.2)
OAC alone, *n* (%)	3137 (2.2)	80 (3.6)
DAPT clopidogrel, *n* (%)	73 730 (50.6)	940 (42.8)
DAPT ticagrelor/prasugrel, *n* (%)	35 381 (24.3)	552 (25.1)
Combination therapy (APT + OAC), *n* (%)	9229 (6.3)	205 (9.3)

APT, antiplatelet therapy; CABG, coronary artery bypass grafting; COPD, chronic obstructive pulmonary disease; CRP, C-reactive protein; DAPT, dual antiplatelet therapy; HF, heart failure; IQR, interquartile range; LEAD, lower extremity artery disease; MI, myocardial infarction; NSAID, non-steroidal anti-inflammatory drug; OAC, oral anticoagulant; PCI, percutaneous coronary intervention; SAPT, single antiplatelet therapy; STEMI, ST-segment elevation myocardial infarction; UGIB, upper gastrointestinal bleeding.

The proportion of missing data was zero or very low for all baseline and treatment variables, except for weight, smoking, and laboratory variables (Supplementary material online, *Table S5*).

### Associated outcomes

**Figure 1:**
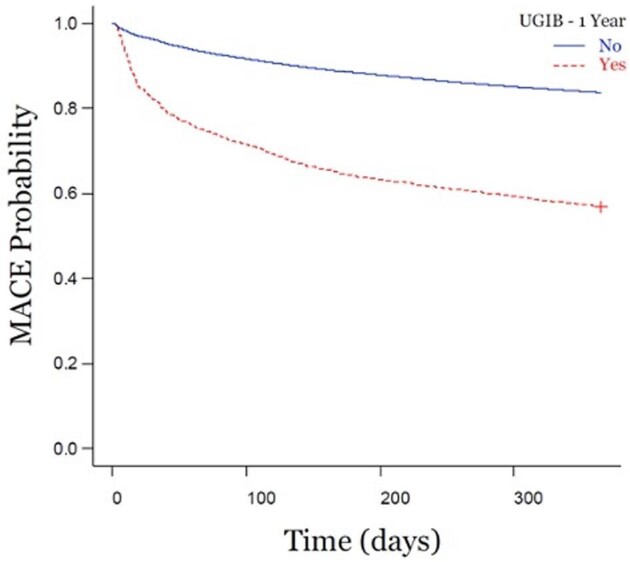
Unadjusted incidence of major adverse cardiovascular events (defined as composite of myocardial infarction, stroke, and all-cause death) in patients with or without upper gastrointestinal bleeding using upper gastrointestinal bleeding as a time-dependent event.

Unadjusted incidence of MACE in patients with or without a UGIB event is shown in *Figure 1*. After adjustment for baseline characteristics and invasive and medical treatment at discharge, UGIB was associated with a two-fold increased risk of MACE (HR 2.00, 95% CI 1.81–2.20). When considering the individual components of MACE separately, UGIB was significantly associated with an increased risk of stroke (HR 1.80, 95% CI 1.32–2.45) and all cause-death (HR 2.86, 95% CI 2.58–3.16), while there was no significant association with recurrent MI (HR 1.17, 95% CI 0.97–1.42) (*Table 2*).

**Table 2:** Crude and adjusted hazard ratios for associated outcomes of upper gastrointestinal bleeding

	Crude HR (95% CI)	Adjusted HR (95% CI)
MACE	2.73 (2.47–3.01)	2.00 (1.81–2.20)
All-cause death	4.23 (4.01–4.88)	2.86 (2.58–3.16)
MI	1.44 (1.90–1.74)	1.17 (0.97–1.42)
Stroke	2.27 (1.69–3.06)	1.80 (1.32–2.45)

CI, confidence interval; HR, hazard ratio; MACE, major adverse cardiovascular event; MI, myocardial infarction.

### Predictors of upper gastrointestinal bleeding

**Figure 2:**
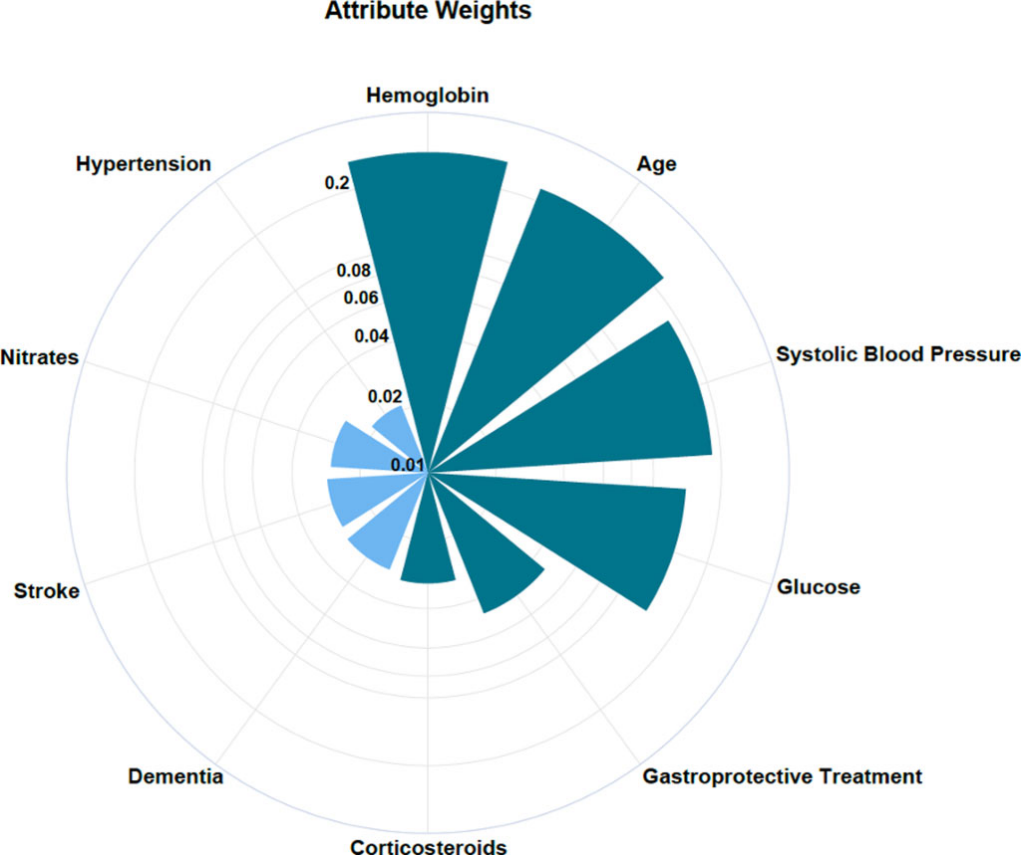
The most important predictors in the best-performing machine learning model, the random forest. For each of the 10 variables, a variable importance weight measure is presented, which is proportional to the increase in the misclassification rate of the random forest, if the variable was removed from the model. Higher importance weights indicate that the variable is more important when predicting upper gastrointestinal bleeding events.

The top six predictors of UGIB in the logistic regression model were haemoglobin, age, previous UGIB, smoking status, antithrombotic treatment, and gastroprotective treatment. Smoking status included three categories: never, former, and active smokers. With never smoker as reference, former and active smokers were associated with increased risk of UGIB. Antithrombotic treatment included five categories: SAPT, OAC alone, DAPT clopidogrel, DAPT ticagrelor/prasugrel, and combination therapy (APT + OAC). With SAPT as reference, combination therapy, DAPT ticagrelor/prasugrel, and OAC alone were associated with increased risk of UGIB, while there was no significant association for DAPT clopidogrel (*Table 3*). The ML models identified both similar and additional predictors: haemoglobin, age, systolic blood pressure, blood glucose, gastroprotective treatment, and corticosteroid treatment (*Figure 2*). The full logistic regression model with 25 predictors (Supplementary material online, *Table S6*) had a *C*-index of 0.67 as compared with the best-performing ML model (random forest) with only 10 predictors and a *C*-index of 0.73 (Supplementary material online, *Figure S3*).

**Figure 3:**
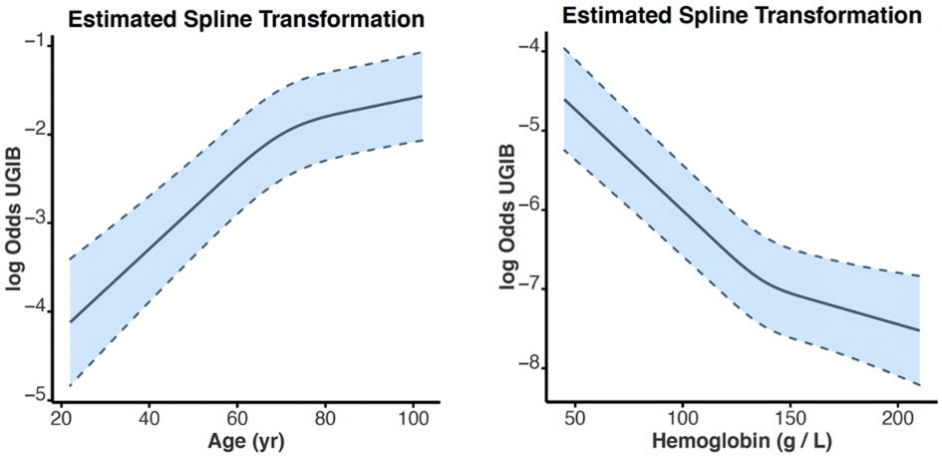
The most important continuous predictors in the logistic regression model. Estimated spline transformation in relation to log odds of upper gastrointestinal bleeding.

**Table 3:** Most important predictors of upper gastrointestinal bleeding in the logistic regression model

Predictor	Odds ratio	Wald $\chi $^2^	Significance
Haemoglobin	NA^a^	241	<0.001
Age	NA^a^	122.3	<0.001
Previous UGIB	2.58	117.6	<0.001
Smoking status		90.8	
Never	Reference		
Active	1.84		<0.001
Former	1.29		<0.001
Antithrombotic treatment		61.0	
SAPT	Reference		
Combination therapy (APT + OAC)	1.56		<0.001
OAC alone	1.52		0.001
DAPT ticagrelor/prasugrel	1.41		<0.001
DAPT clopidogrel	1.03		0.711
Gastroprotective treatment	1.33	37.4	<0.001

The six most important predictors of UGIB with corresponding Wald $\chi $^2^ values, odds ratios, and *P*-values. Smoking status has three categories and antithrombotic treatment has five categories. APT, antiplatelet therapy; DAPT, dual antiplatelet therapy; OAC, oral anticoagulant; SAPT, single antiplatelet therapy; UGIB, upper gastrointestinal bleeding.

^a^See illustration in *Figure 3*.

## Discussion

This nationwide observational registry-based cohort study found a 1-year incidence of UGIB of 1.5% and an associated increase in mortality and stroke. The most important predictors of UGIB, when combining the results of the logistic regression and ML models, were haemoglobin, age, systolic blood pressure, blood glucose, previous UGIB, smoking status, antithrombotic treatment, gastroprotective treatment, and corticosteroid treatment.

### Incidence and associated outcomes

In relation to the bleeding incidence previously reported from the SWEDEHEART registry,^16^ a 1-year incidence of UGIB of 1.5% constitutes approximately one-third of all out-of-hospital bleeding events. These novel insights substantiate data from previous studies,^6,17^ showing that UGIB is a common and feared complication with substantial consequences in terms of morbidity, mortality, and medical care costs.

To our knowledge, our study is the largest on UGIB following MI in unselected patients, also including individuals treated with OAC and those receiving a primary non-invasive treatment strategy. OAC therapy increases bleeding risk significantly especially in combination with APT and in particular the NOACs are known to increase the risk of GI bleeding.^18^ Approximately 10–15% of all MI patients have indication for OAC and the use of OAC in addition to APT has been increasing over the last decade after the introduction of the NOACs.^16^ Even if PCI is not performed, the recommended treatment strategy for patients with acute MI is similar to DAPT using a potent P2Y12 inhibitor for up to 12 months.^1,2^ Therefore, when evaluating out-of-hospital bleeding, it is essential to also include these conservatively treated patients who may often be at even higher risk of bleeding than patients undergoing PCI due to a higher comorbidity burden.

While most previous studies on associated prognosis have included both upper and lower GI bleedings, our study is the first large study investigating the associated prognosis of UGIB after MI. GI bleeding, including both upper and lower origins, was associated with increased mortality in different settings, ranging from PCI registry data^19^ or a STEMI cohort^11^ to a post-hoc analysis of acute coronary syndrome patients with moderate to high risk from the ACUITY (Acute Catheterization and Urgent Intervention Triage strategY) trial.^12^ Overall, our results are consistent and extend the evidence of UGIB to a nationwide cohort of unselected patients with MI.

### Possible mechanisms linking upper gastrointestinal bleeding with mortality

In the fully adjusted analysis of associated outcomes, the strongest association was for mortality, with a nearly three-fold increased risk. The mechanisms linking UGIB with mortality are probably multifactorial. While the most severe bleedings can cause direct life-threatening situations, the consequences of less severe bleedings are indirect. For example, massive UGIB can cause haemodynamic compromise resulting in death. Blood transfusion may exert indirect effects by causing systemic inflammation with a prothrombotic state, increased oxidative stress, and paradoxically decreased oxygen delivery that all could contribute to worse outcomes.^20^ Even a mild bleeding not requiring blood transfusion may lead to discontinuation of antithrombotic treatment and thus indirectly affect prognosis.

### Different methods to identify predictors

Logistic regression is a well-established method to identify predictors in clinical settings with a reasonable number of baseline predictors and it is rather easily interpreted. However, the method has limited capacity to handle large number of variables or complex interactions and/or non-linear relationships. We therefore added ML methods agnostic to traditional assumptions about the data with the potential to appreciate complex interactions and non-linearities in addition to the classical logistic regression method. The best-performing ML model, random forest, did indeed show better discrimination than the logistic regression model.

### Predictors of upper gastrointestinal bleeding

Our aim in this study was not to derive a novel prediction model but to explore and describe the potential predictors of UGIB. We found that many of the predictors of UGIB were similar to well-known predictors of all-cause spontaneous bleeding, such as low haemoglobin, previous bleeding, high age, and more intensive antithrombotic treatment. This is not surprising since UGIB constitutes a significant proportion of all spontaneous bleedings.^5^ Corticosteroids are known to increase risk of UGIB by negative effect on the gastric mucosa. Systolic blood pressure is a predictor in the CRUSADE (Can Rapid risk stratification of Unstable angina patients Suppress ADverse outcomes with Early implementation of the ACC/AHA guidelines),^21^ which predicts in-hospital bleeding. Gastroprotective treatment was associated with increased risk of UGIB. This could perhaps partly be explained by confounding by indication; i.e. patients with high bleeding risk, already established ulcer or gastric disease, or previous UGIB are more often treated with gastroprotective drugs.

Smoking has long been well established as an ischaemic risk factor but has not previously been considered to increase bleeding risk. For example, in the DAPT score, cigarette smoking was one of the ischaemic predictor variables.^22^ However, in the logistic regression model in our study, smoking status was one of the strongest predictors of UGIB. A common link could be an active, concomitant *H. pylori* infection that in combination with antithrombotic therapy substantially increases the risk for UGIB and is proposed to be associated with smoking.^23^

The random forest ML model also included glucose among the top four predictors of UGIB, although this parameter has previously not been thoroughly investigated in this field. A potential explanation of this phenomenon could be that glucose level is a proxy for the degree of stress that, in turn, could influence UGIB incidence.

### Clinical relevance

Given the prognostic consequences of both ischaemic and bleeding complications, the optimal treatment strategy has to balance the risk of these events.^24^ There are now many alternatives for such individualized approach, but it is still unclear how to best stratify these risks. Several scores have been developed for out-of-hospital bleeding risk assessment^22,25,26^ and recently criteria by the Academic Research Consortium for High Bleeding Risk^27^ have been proposed.

In addition to the well-known risk factors for major bleeding, the results of our study suggest the existence of further specific predictors useful in risk stratifying UGIB patients, such as blood glucose, smoking status, and previous UGIB.

If patients with high risk of UGIB could be identified, there are several prophylactic measures to lower the risk of UGIB. First, general approaches that lower risk of bleeding probably also lower the risk of UGIB. Individualized therapy with shorter DAPT and de-escalation to a less potent P2Y12 inhibitor^28^ may lower bleeding overall, while aspirin-free strategies^8,9^ may not only lower bleeding overall but also offer a direct mechanism to reduce the negative effect on the gastric mucosa of cyclooxygenase inhibition by acetylsalicylic acid.^29^ Second, there are specific therapies to prevent UGIB by use of PPIs^6,7^ or other gastroprotective drugs and test-and-treat strategies for active *H. pylori*.^30^ Controversy remains concerning the risks associated with long-term use of PPIs, including pneumonia, dementia, cardiovascular events, and impaired renal function,^6^ but nevertheless the use of PPIs has increased over the last decade.^31^ Despite clear guideline recommendations for *H. pylori* testing,^10^ this approach is not yet implemented in clinical practice, possibly due the lack of data from large-scale randomized clinical trials.

### Limitations

Inherent to the observational design, we were limited by the information available in our registry and we did not have data on relevant information such as alcohol intake, previous known peptic ulcer, gastro-oesophageal reflux disease, *H. pylori* infection, or dyspeptic disease. Thus, lack of important predictor variables could perhaps partly explain why the logistic regression model and the best-performing ML model did not have higher discriminative capacity. Even though we adjusted for baseline characteristics, invasive treatment, and medical treatment at discharge, there may be residual confounding biasing the associated risk of adverse events.

## Conclusions

During the first year after acute MI, readmission because of UGIB is common and significantly associated with poor prognosis. By the use of ML techniques in addition to traditional logistic regression, beyond the well-known predictors of major bleeding, new predictors of UGIB such as blood glucose and smoking status were identified.

## Supplementary Material

pvab059_Supplemental_File
