# Supplementary material for: Incidence, associated outcomes, and predictors of upper gastrointestinal bleeding following acute myocardial infarction: a SWEDEHEART-based nationwide cohort study
Source: Eur Heart J Cardiovasc Pharmacother. 2021 Aug 23;8(5):483–91. doi: 10.1093/ehjcvp/pvab059 (PMC9366628; doi:10.1093/ehjcvp/pvab059)
Supplement: pvab059_Supplemental_File [file pvab059_supplemental_file.docx]

**Supplemental appendix**

**To:**

**Incidence, associated outcomes and predictors of upper gastrointestinal bleeding following acute myocardial infarction – a SWEDEHEART based nationwide cohort study**

Philip Sarajlic, M.D.^1*^; Moa Simonsson, M.D.^2,3 *^; Tomas Jernberg, M.D., Ph.D.^2^; Magnus Bäck, M.D., Ph.D.^1,3^; and Robin Hofmann, M.D., Ph.D.^4^

1. Department of Medicine, Karolinska Institute, Stockholm, Sweden.
2. Department of Clinical Sciences, Cardiology, Karolinska Institute, Danderyd Hospital, Stockholm, Sweden
3. Theme Heart and Vessels, Division of Valvular and Coronary Disease, Karolinska University Hospital, Stockholm, Sweden.
4. Department of Clinical Science and Education, Division of Cardiology, Karolinska Institute, Sodersjukhuset, Stockholm, Sweden

* shared first author

**Table of contents**

| Figure 1 Consort diagram | p 3 |
| --- | --- |
| Figure 2 Spline transformation of the continuous variables vs log Odds of UGIB | p 4 |
| Figure 3 Model Comparison by ROCs | p 5 |
| Table 1 Upper Gastrointestinal bleeding ICD-10 diagnoses | p 6 |
| Table 2a Previous bleeding defined as any hospitalisation with bleeding ICD-9 or ICD-10 code as listed below in the NPR before the index MI | p 7 |
| Table 2a Previous upper gastrointestinal bleeding defined as any hospitalisation with upper gastrointestinal bleeding ICD-9 or ICD-10 code as listed below in the NPR before the index MI. | p 8 |
| Table 3 Predictor variables in the logistic regression model and variables in the Cox regression models | p 9 |
| Table 4 Predictor variables in the ML models (n=105) | p 10 |
| Table 5 Proportion of missing values in Table 1 variables | p 11 |
| Table 6 Full logistic regression model | p 13 |
| Statistical Methods | p 14 |

**Figure S1 Consort diagram**

**
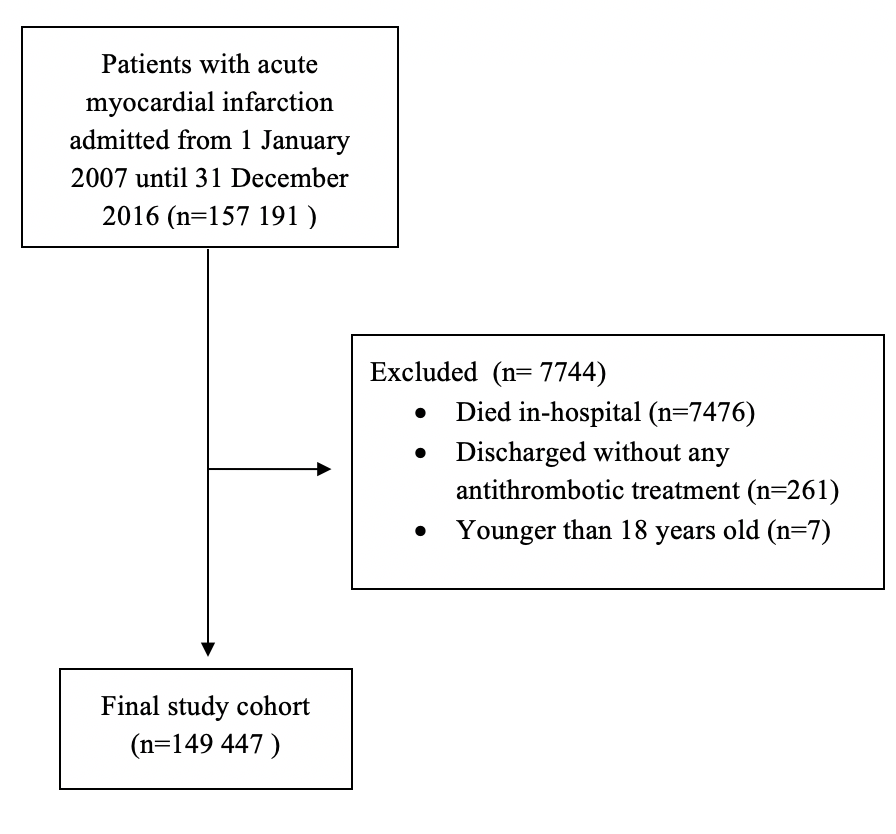
**

**Figure S2 Spline transformation of the continuous variables vs log Odds of UGIB**

**
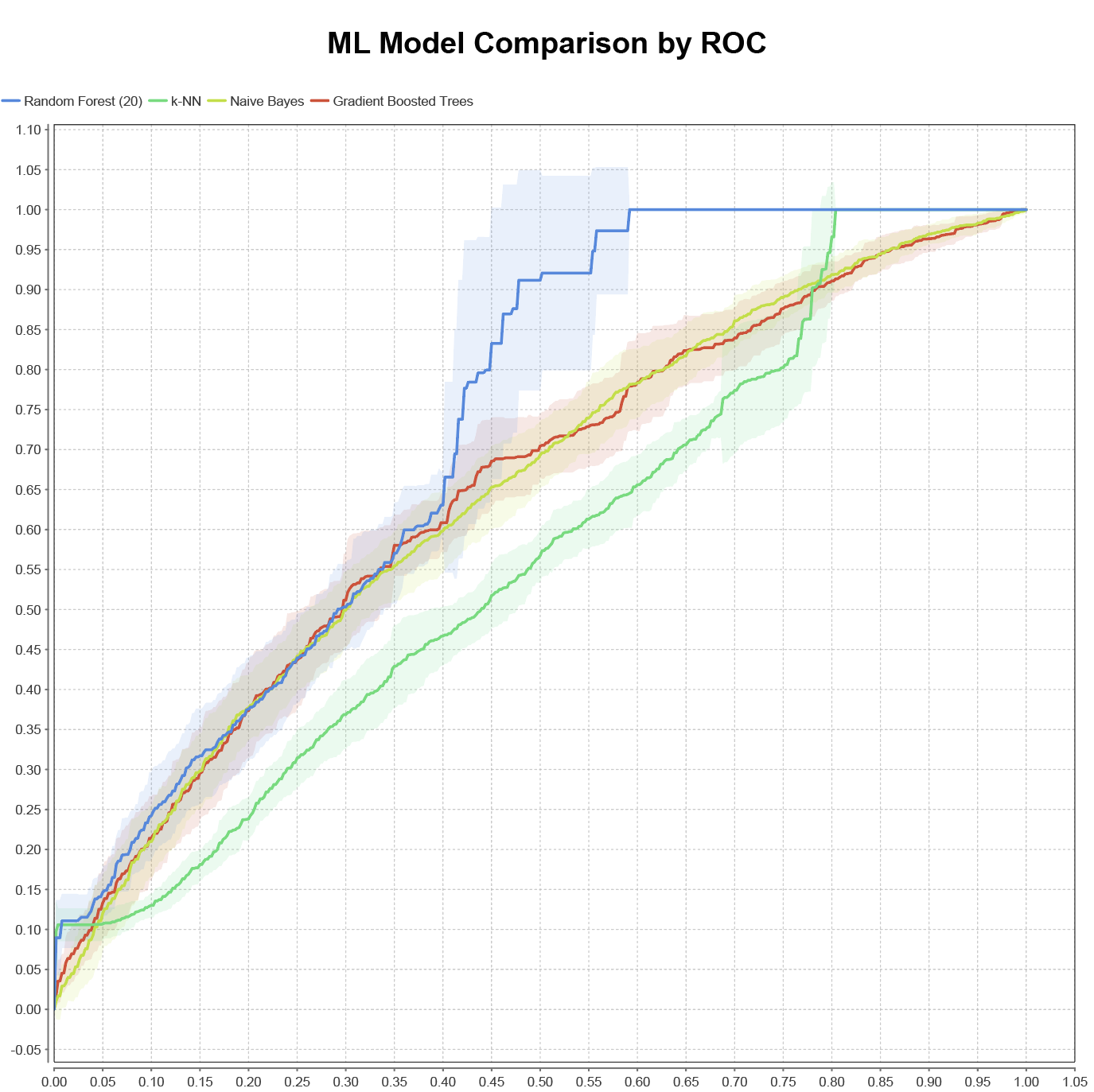
Figure S3 Model Comparison by ROCs**


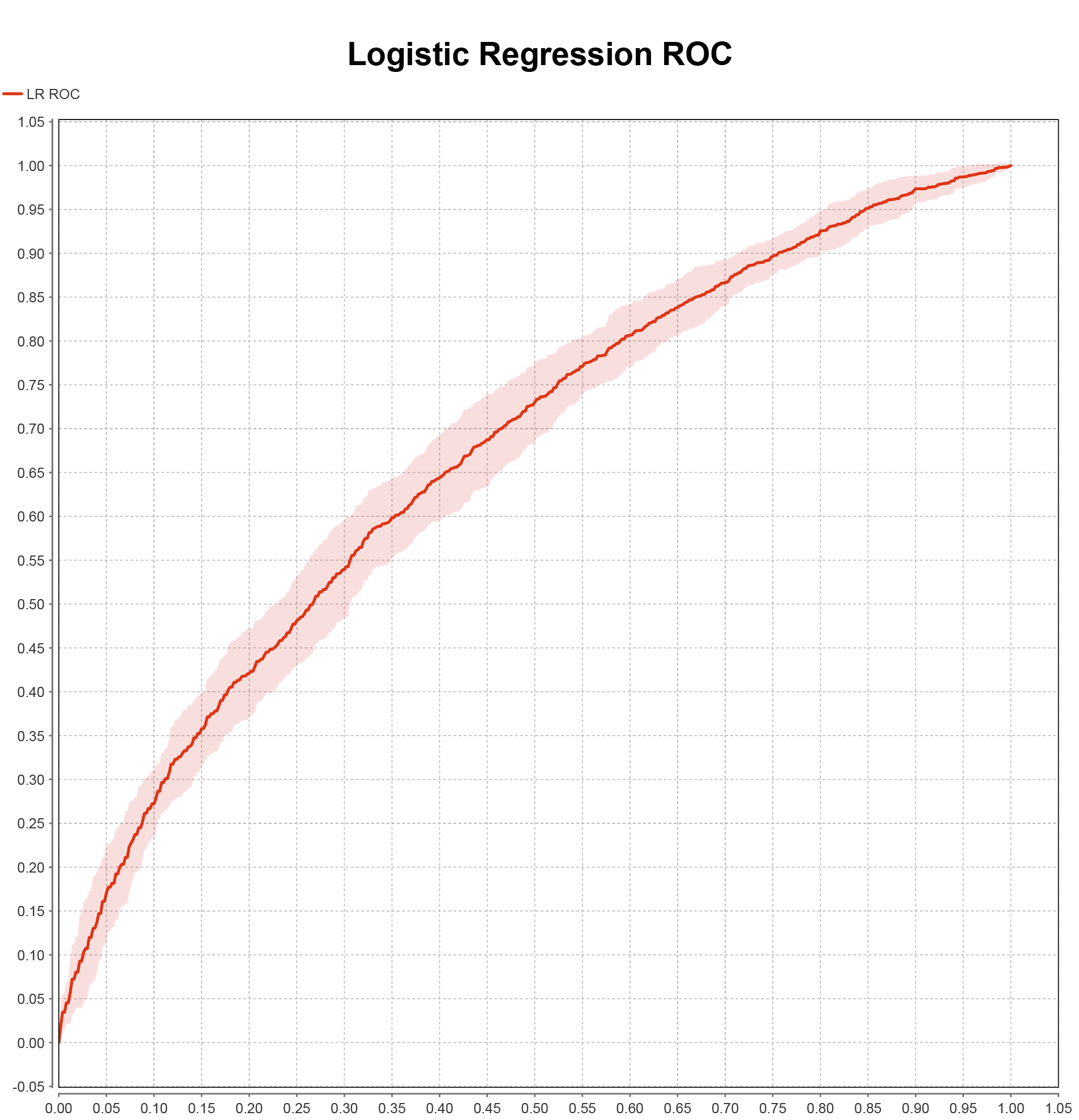


**Table 1 Upper Gastrointestinal bleeding ICD-10 diagnoses**

| **ICD-10 code** |  |  |  |
| --- | --- | --- | --- |
| K250 | Acute gastric ulcer with haemorrhage | |  |
| K252 | Acute gastric ulcer with both haemorrhage and perforation | |  |
| K254 | Chronic or unspecified gastric ulcer with haemorrhage | |  |
| K256 | Chronic or unspecified gastric ulcer with both haemorrhage and perforation | |  |
| K260 | Acute duodenal ulcer with haemorrhage | |  |
| K262 | Acute duodenal ulcer with both haemorrhage and perforation | |  |
| K264 | Chronic or unspecified duodenal ulcer with haemorrhage | |  |
| K266 | Chronic or unspecified duodenal ulcer with both haemorrhage and perforation | |  |
| K270 | Acute peptic ulcer, site unspecified, with haemorrhage | | |
| K272 | Acute peptic ulcer, site unspecified, with both haemorrhage and perforation | | |
| K274 | Chronic or unspecified peptic ulcer, site unspecified with haemorrhage | | |
| K276 | Chronic or unspecified peptic ulcer, site unspecified with both haemorrhage and perforation | | |
| K280 | Acute gastrojejunal ulcer with haemorrhage | | |
| K282 | Acute gastrojejunal ulcer with both haemorrhage and perforation | | |
| K284 | Chronic or unspecified gastrojejunal ulcer with haemorrhage | | |
| K286 | Chronic or unspecified gastrojejunal ulcer with both haemorrhage and perforation | | |
| K290 | Acute gastritis with bleeding | |  |
| K922 | Gastrointestinal haemorrhage, unspecified |  |  |

**Table 2a Previous bleeding defined as any hospitalisation with bleeding ICD-9 or ICD-10 code as listed below in the NPR before the index MI**

| **ICD-9** | **ICD-10** |  |
| --- | --- | --- |
| 285B | D629 | Anaemia following acute significant bleeding |
|  | D500 | Iron deficiency anaemia following chronic blood loss |
|  | H356, H431, H450 | Ocular bleeding |
|  | H922 | Bleeding from the ear |
| 430 , 431 , 432 | I60, I61, I62 | Cerebral and intracranial bleeding |
| 456A | I850 | Oesophageal varices with bleeding |
| 531A, 531C, 531E, 531G, 532A, 532C, 532E, 532G, 533A, 533C, 533E, 533G, 534A, 534C, 534E, 534G, | K250, K252, K254, K256,  K260, K262, K264, K266,  K270, K272, K274, K276,  K280, K282, K284, K286, | Ulcus with bleeding |
| 530H | K226 | Mallory-Weiss syndrom |
|  | K290 | Acute gastritis with bleeding |
| 569D | K625 | Bleeding in anus or rectum |
| 578 | K920 | Hematemesis |
|  | K921 | Melena |
|  | K922 | Gastrointestinal haemorrhage, unspecified |
|  | N421, N501, N938, N939, N950, R319 | Urogenital bleeding |
|  | R041, R042, R048, R049 | Airway bleeding |
|  | T810 | Secondary bleeding or hematoma complicating surgery or medical interventions |

**Table 2a Previous upper gastrointestinal bleeding defined as any hospitalisation with upper gastrointestinal bleeding ICD-9 or ICD-10 code as listed below in the NPR before the index MI**

| **ICD-9** | **ICD-10**  **code** |  |  |  |
| --- | --- | --- | --- | --- |
| 531A | K250 | Acute gastric ulcer with haemorrhage | |  |
| 531C | K252 | Acute gastric ulcer with both haemorrhage and perforation | |  |
| 531E | K254 | Chronic or unspecified gastric ulcer with haemorrhage | |  |
| 531G | K256 | Chronic or unspecified gastric ulcer with both haemorrhage and perforation | |  |
| 532A | K260 | Acute duodenal ulcer with haemorrhage | |  |
| 532C | K262 | Acute duodenal ulcer with both haemorrhage and perforation | |  |
| 532E | K264 | Chronic or unspecified duodenal ulcer with haemorrhage | |  |
| 532G | K266 | Chronic or unspecified duodenal ulcer with both haemorrhage and perforation | |  |
| 533A | K270 | Acute peptic ulcer, site unspecified, with haemorrhage | | |
| 533C | K272 | Acute peptic ulcer, site unspecified, with both haemorrhage and perforation | | |
| 533E | K274 | Chronic or unspecified peptic ulcer, site unspecified with haemorrhage | | |
| 533G | K276 | Chronic or unspecified peptic ulcer, site unspecified with both haemorrhage and perforation | | |
| 534A | K280 | Acute gastrojejunal ulcer with haemorrhage | | |
| 534C | K282 | Acute gastrojejunal ulcer with both haemorrhage and perforation | | |
| 534E | K284 | Chronic or unspecified gastrojejunal ulcer with haemorrhage | | |
| 534G | K286 | Chronic or unspecified gastrojejunal ulcer with both haemorrhage and perforation | | |
|  | K290 | Acute gastritis with bleeding | |  |
| 578 | K922 | Gastrointestinal haemorrhage, unspecified |  |  |

**Table 3 Predictor variables in the logistic regression model and variables in the Cox regression models**

|  |  |
| --- | --- |
| Demographics | Gender |
|  | Age |
|  | Weight |
|  | STEMI |
| Procedural characteristics | PCI in hospital |
|  | CABG in hospital |
| Medical history | Current smoking |
|  | Hypertension |
|  | Diabetes |
|  | Previous MI |
|  | Previous PCI |
|  | Previous CABG |
|  | Previous CHF |
|  | Previous stroke |
|  | Previous LEAD |
|  | Previous UGIB |
|  | Previous Cancer |
|  | Previous COPD |
| Laboratory parameters | Haemoglobin |
|  | Creatinine |
|  | CRP |
| Discharge medication | Gastro protective treatment |
|  | Corticosteroid |
|  | NSAID |
| Antithrombotic treatment at discharge | SAPT |
|  | OAC alone |
|  | DAPT clopidogrel |
|  | DAPT ticagrelor or prasugrel |
|  | Combination therapy (APT + OAC) |

APT: antiplatelet therapy, CABG: coronary artery bypass grafting, COPD: chronic obstructive pulmonary disease, CRP: C-reactive protein, DAPT: dual antiplatelet therapy, HF: heart failure, LEAD: lower extremity artery disease MI: myocardial infarction, NSAID: non-steroidal anti-inflammatory drug, OAC: oral anticoagulant, PCI: percutaneous coronary intervention, SAPT: single antiplatelet therapy, STEMI: ST-segment elevation myocardial infarction, UGIB: upper gastrointestinal bleeding

**Table 4 Predictor variables in the ML models (n=105)**

DEMOGRAPHICS TREATMENT ON ADMISSION:

| Myocardial infarction type (ICD 10 code I 21 or I 22)  Age  Sex  Height  Weight  PREHOSPITAL  Arrival with ambulance (No, Yes to ER, Yes directly to cath lab or CCU, unknown)  CPR before hospital  STATUS ON ARRIVAL  ECG rythm  ECG STT changes  STEMI  Cardiac shock  Pulmonary rales  Heartrate  Systolic blood pressure  Diastolic blood pressure  Presenting symptoms: (Chestpain, dyspnea, Cardiac arrest, other, unknown)  Occupation status  Smoking status  Snuff status  LABORATORY TESTS ON ARRIVAL  Haemoglobin  Creatinine  Cholesterol total  HDL cholesterol  LDL cholesterol  Triglycerides  Glucose  CRP  MEDICAL HISTORY AS RECORDED IN SWEDEHEART  Diabetes  Hypertension  Previous MI  Previous PCI  Previuos cardiac surgery  History of HF  Previous stroke  MEDICAL HISTORY AS RECORDED IN THE NATIONAL PATIENT REGISTER  Diabetes  Hypertension  Previous MI  Previous PCI  Previous cardiac surgery  History of HF  Previous stroke  Previous LEAD  Previous UGIB  Previous bleeding  Renal disease  Dialysis  COPD  Previous cancer  Dementia | Oral anticoagulants  Antiplatelet P2Y12 inhibitor  Aspirin  Beta blockers  Calcium antagonist  Diabetes insulin  Diabetes drugs oral  Digitalis  Diuretics  Statins  Ezetimibe  Nitrates  TREATMENT AND EVALUTION IN-HOSPITAL  Reperfusion treatment (No, thrombolysis, primary PCI, acute CABG, Acute coronary angio but no further intervention)  Anticoagulants  Glycoprotein IIb/IIIa blocker  Beta blockers  Diuretics  Inotropes  Nitrates  Left ventricular function (Normal (≥50%), Slightly depressed (40-49%), Moderately depressed (30-39%), Severely depressed (<30%), Unknown  CABG (no, Yes acute, Yes during hospital stay, Yes planned after discharge)  Electrical_devices (No, Pacemaker, ICD, CRT, CRT-D, unknown)  CPAP  BiomarkerCombined (Troponin Hs troponin and CKMB)  OUTCOMES IN-HOSPITAL  Reinfarction  Bleeding in-hospital  Resuscitated cardiac arrest  Cardiogenic shock  AV block  New Atrial fibrillation or flutter  TREATMENT AT DISCHARGE:  Oral anticoagulants  Antiplatelet P2Y12 inhibitor  Aspirin  Beta blockers  Calciumantagonist  Diabetes insulin  Diabetes drugs oral  Digitalis  Diuretics  Statins  Ezetimibe  Nitrates  RAAS blocker (renin-angiotensin or angiotensin II receptorblocker )  OTHER TREATMENT  Gastroprotective drugs 6 mo before  Gastroprotective drugs 2 weeks after  Corticosteroid treatment 6 mo before  Corticosteroid treatment 2 weeks after  NSAID 6 mo before  NSAID 2 weeks after  OTHER VARIABLES  ECG rythm at discharge  Blood glucose measured (No/Yes)  Coronary angio (No/yes)  PCI (No/Yes)  CABG (No/yes)  Rescue PCI No/yes)  Hospital stay in days |
| --- | --- |

APT: antiplatelet therapy, CABG: coronary artery bypass grafting, COPD: chronic obstructive pulmonary disease, CPAP: continuous positive airway pressure, CRP: C-reactive protein, DAPT: dual antiplatelet therapy, ECG: electrocardiogram, HF: heart failure, LEAD: lower extremity artery disease MI: myocardial infarction, NSAID: non-steroidal anti-inflammatory drug, OAC: oral anticoagulant, PCI: percutaneous coronary intervention, RAAS: renin-angiotensin aldosterone system, SAPT: single antiplatelet therapy, STEMI: ST-segment elevation myocardial infarction, UGIB: upper gastrointestinal bleeding

**Table 5 Proportion of missing values in Table 1 variables**

|  | % Missing |
| --- | --- |
| Age | 0 |
| Sex | 0 |
| Weight | 5.5 |
| Smoking | 7.2 |
| Previous UGIB | 0 |
| CRP | 10.9 |
| Creatinine | 4.1 |
| Diabetes | 0 |
| Hypertension | 0 |
| Previous HF | 0 |
| Previous MI | 0 |
| Previous CABG | 0 |
| Previous PAD | 0 |
| Previous COPD | 0 |
| Previous Cancer | 0 |
| Gastroprotective Treatment | 0 |
| Corticosteroids | 0 |
| NSAID | 0 |
| Hemoglobin | 6.2 |
| Aspirin | 0.02 |
| Oral anticoagulants | 0.08 |
| Other Antiplatelet Drugs | 0.03 |
| Hospital STEMI | 0 |
| Hospital PCI | 0 |
| Previous PCI | 0 |
| Previous Stroke | 0 |
|  |  |

**Table S6 Full logistic regression model**

|  | Wald$\boldsymbol{\chi}$^2^ | Exp(B) (95% CI) | Significance |
| --- | --- | --- | --- |
| Age | **122.3** | N/A* | 0.000 |
| Sex male | 6.0 | 0.88 (0.79-0.96) | 0.015 |
| Weight | 11.2 | N/A* | 0.005 |
| STEMI | 4.4 | 0.90 ( 0.81-0.99) | 0.036 |
|  |  |  |  |
| PCI in hospital | 5.3 | 1.14 (1.02-1.28) | 0.021 |
| CABG in hospital | 0.0 | 0.99 (0.77-1.27) | 0.029 |
| Smoking status  Never smoker  Former smoker  Active smoker | **90.8**  Ref  23.0  90.5 | 1.29 (1.16-1.42)  1.84 (1.62-2.09) | 0.000  0.000 |
| Hypertension | 19.5 | 1.25 (1.13-1.37) | 0.000 |
| Diabetes | 0.1 | 0.99 (0.89-1.09) | 0.782 |
| Previous MI | 11.2 | 0.81 (0.72-0.92) | 0.001 |
| Previous PCI | 0.6 | 0.94 (0.81-1.10) | 0.429 |
| Previous CABG | 4.8 | 0.83 (0.70-0.98) | 0.029 |
| Previous HF | 1.2 | 1.08 (0.94-1.23) | 0.277 |
| Previous stroke | 0.2 | 0.98 (0.86-1.10) | 0.689 |
| Previous LEAD | 2.2 | 1.12 (0.97-1.31) | 0.134 |
| Previous UGIB | **117.6** | 2.58 (2.17-3.06) | 0.000 |
| Previous Cancer | 3.0 | 1.18 (0.98-1.43) | 0.083 |
| Previous COPD | 8.4 | 1.23 (1.07-1.41) | 0.004 |
| Haemoglobin | **241.0** | N/A* | 0.000 |
| Creatinine | 14.1 | N/A* | 0.000 |
| CRP | 0.0 | N/A* | 0.955 |
| Gastro protective treatment | **37.4** | 1.33 (1.21-1.45) | 0.000 |
| Corticosteroid | 2.7 | 1.17 (0.97-1.40) | 0.103 |
| NSAID | 0.4 | 1.10 (0.82-1.48) | 0.525 |
| Antithrombotic treatment | **61** |  |  |
| SAPT | ref |  |  |
| OAC alone | 11.1 | 1.52 (1.19-1.95) | 0.001 |
| DAPT clopidogrel | 0.137 | 1.03 (0.88-1.17) | 0.711 |
| DAPT ticagrelor or  prasugrel | 18.8 | 1.41 (1.21-1.65) | 0.000 |
| Combination therapy  (APT + OAC) | 22.6 | 1.56 (1.30-1.87) | 0.000 |

*spline transformations of the continuous variables vs logodds of UGIB are illustrated graphically in Figure S2

APT: antiplatelet therapy, CABG: coronary artery bypass grafting, COPD: chronic obstructive pulmonary disease, CRP: C-reactive protein, DAPT: dual antiplatelet therapy, , HF: heart failure, LEAD: lower extremity artery disease MI: myocardial infarction, NSAID: non-steroidal anti-inflammatory drug, OAC: oral anticoagulant, PCI: percutaneous coronary intervention, SAPT: single antiplatelet therapy, STEMI: ST-segment elevation myocardial infarction, UGIB upper gastrointestinal bleeding

**Statistical Methods**

To further explore the association between clinical variables collected in the SWEDEHEART patient cohort and upper gastrointestinal bleeding (UGIB), we trained and validated four machine learning (ML) models. To account for the imbalance in the number of patients having/not having a UGIB event during the first year of follow-up, non bleeders were downsampled, yielding a dataset of 5116 patients that was used to train and validate the ML models. To ensure that a representative sample of the non-bleeders was taken, we compared the results from descriptive statistics and logistic regression to ascertain whether the coefficients were going in the same direction as in the LR model using data from the whole population. The ML models were tasked with correctly classifying whether patients had at least one UGIB event during one year of follow-up. The classifier algorithms of choice, using default model parameters, were: Naïve Bayes, k-Nearest Neighbor, Gradient Boosted Trees, and Random Forest. The models were evaluated after performing k - fold cross-validation, which is a technique that allows for algorithm comparison by partitioning samples into k folds and iteratively estimating model performance. More specifically, each model is trained on k – 1 folds and then validated on the remaining fold. This procedure is repeated until all folds have been used for validation once and average performance across all folds is reported. In this study, a k value of 10 was chosen.

To compare the classification performance of each model, ROC curves were plotted, allowing for determination of the area under the curve (AUC) for each classifier. Weights for variable importance of the model with the highest AUC were further investigated and illustrated in figure 1. Each weight is proportional to the amount of patients that would get misclassified if a certain variable was removed from the model. Thus, higher weight values indicate higher variable importance in regards to predicting the outcome. To avoid contamination and data leakage, predictor selection was performed as a separate process inside the training folds, enhancing the external generalizability of the results. Since this approach can be very computationally demanding, especially in datasets with a large number of samples, the evolutionary feature selection algorithm process was chosen since it achieves a good balance between finding the most relevant variables and computational complexity. A nested split validation process containing a random forest model was chosen as the main subprocess for the feature selection algorithm to evaluate its performance after a 0.7 split ratio. All ML models were created and assessed in RapidMiner Studio 9.8 (RapidMiner, Inc 2020).
